# Multifractal Company Market: An Application to the Stock Market Indices

**DOI:** 10.3390/e24010130

**Published:** 2022-01-16

**Authors:** Michał Chorowski, Ryszard Kutner

**Affiliations:** Faculty of Physics, University of Warsaw, Pasteur Str. 5, PL-02093 Warsaw, Poland; ryszard.kutner@fuw.edu.pl

**Keywords:** multiscale partition function, multifractal analysis, company market, 89.65 Gh, 05.40.-a, 89.75.Da

## Abstract

Using the multiscale normalized partition function, we exploit the multifractal analysis based on directly measurable shares of companies in the market. We present evidence that markets of competing firms are multifractal/multiscale. We verified this by (i) using our model that described the critical properties of the company market and (ii) analyzing a real company market defined by the S&P500 index. As the valuable reference case, we considered a four-group market model that skillfully reconstructs this index’s empirical data. We point out that a four-group company market organization is universal because it can perfectly describe the essential features of the spectrum of dimensions, regardless of the analyzed series of shares. The apparent differences from the empirical data appear only at the level of subtle effects.

## 1. Introduction

In the last two decades, multifractal properties have been the subject of intense research in very different areas of science [1,2,3,4,5,6,7,8,9,10,11,12,13]. The fashion for searching for new areas of multifractality is still ongoing. The shape, location, and spread of the spectrum of dimensions (singularities)—the leading multifractality indicator—provide invaluable information about the layout. We use the formalism [14] that describes not only systems in the state of statistical equilibrium but also stationary states. Furthermore, we indicate that formalism can easily be extended to transient states.

Our approach is complementary to the commonly used multifractal detrended fluctuation analysis (MF-DFA) [1,2]. More precisely, in the presence of state intervention, our concept of using (normalized) market shares for multifractal analysis of the market of competing firms is new. It starts with a partition function expressed directly by shares. Thanks to this, it bypasses the onerous preparation of traditional MF-DFA, based on a fluctuation function built with the help of time series.

We demonstrate how our method works with the example of a competing company market model published previously [15]. In this model, we assume that companies can merge, create spin-offs, and go bankrupt in the presence of state intervention. This tendency for firms to disappear from the market can counterbalance the tendency to design firms, leading to critical phenomena. We examined these phenomena in our previous work [15]. In this work, we explore a different aspect of the market model of competing companies, namely, multifractality.

Moreover, we show that the actual market of S&P500 companies is multifractal. Finally, we indicate that this market can be (roughly) described by the multifractal formalism, in which companies are divided into four groups differing significantly in market shares.

The paper consists of two parts. The first part consists of Section 1 (Introduction) together with Section 2 (Theory), which on the example of our critical company market model [15] presents the multifractal approach. The second part presents this multifractal approach to the real market of the S&P500 index. Moreover, this part compares the obtained results for the actual market with the four-group market model.

## 2. Theory

### 2.1. Definition of Partition Function

The multifractal behavior of the market of competing firms is a new concept. We based this concept on the characteristic for this market, the partition function given by the formula [14]

(1) $$\begin{array}{c} Z\left(\beta \right)=\sum_{n=1}^{N}\omega_{n}^{\beta }, \end{array}$$

where $\omega_{n}$ is the (normalized) market share of firm *n*, while *N* is the number of firms in the market; both a priori given quantities we can obtain, at a given time, from simulations, empirical data, or from theory.

**Figure 1:**
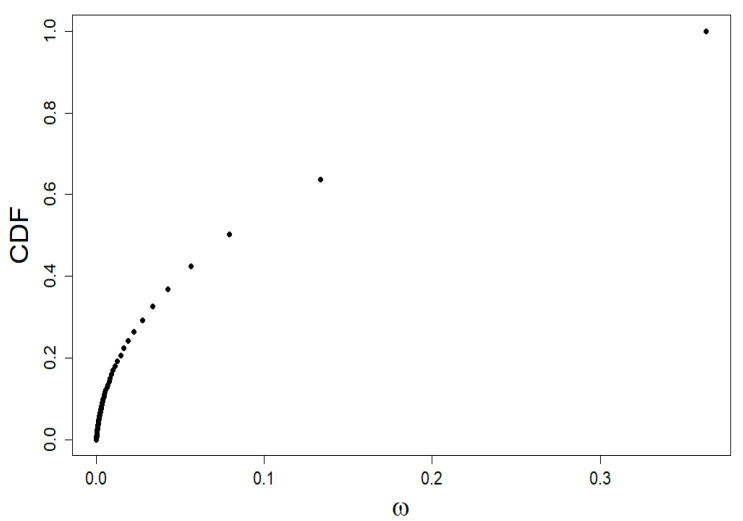
Quetelet curve: the dependence of the standardized rank of companies generated within our model, i.e., CDF, on their shares ω. It is precisely to analyze this simulation data that we use multifractal formalism.

We characterize the market shares of companies using the Quetelet ranking (see Figure 1), i.e., we build a plot of cumulative distribution function (CDF) versus company share value taken from simulation within our model.

The partition function in the form given by Equation (1) is ready to study the multiscale nature of the ω distribution. This multiscale nature comes from the hierarchical distribution of firms’ sizes.

**Figure 2:**
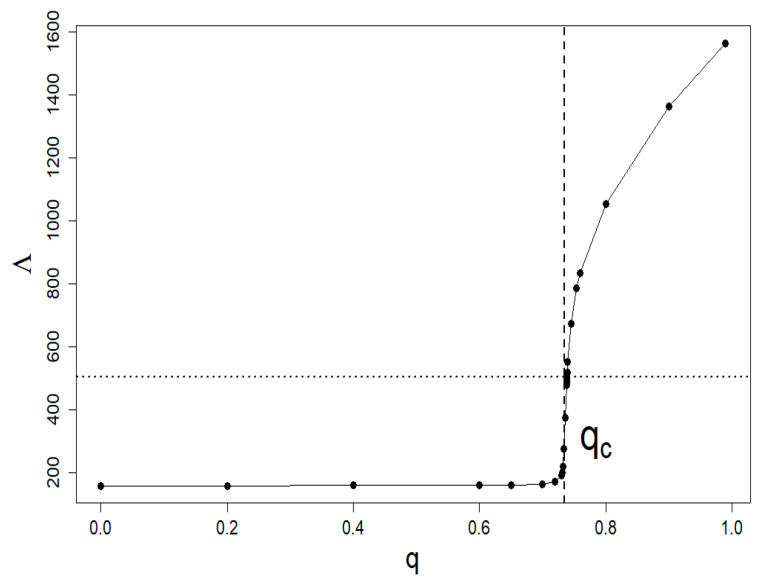
The typical dependence of Λ (=$N_{st}$) vs. interventionism level *q* at fixed η (=0.5) and λ (=0.9). It is a flat phase diagram where a continuous phase transition is clearly visible at $q_{c}$ (=0.734). All other plots in this section have the same η and λ parameters as this plot.

In this section, we limit ourselves to systems in steady states; therefore, we assume that $N=N_{st}$. Recall that in our model $N_{st}$ is clearly related to the level of intervention 0≤q≤1, its effectiveness 0≤η≤1, and the company’s activity 0≤λ≤1 [15]. Figure 2 shows a typical relationship $N_{st}$ vs. *q* with η (=0.5) and λ (=0.9) fixed. The location of the $q_{c}$ criticality threshold is clearly visible, signaling a continuous phase transition.

The partition function, Z(β), obeys two basic properties,

(2) $$\begin{array}{c} Z(\beta =0)=N, \end{array}$$

and

(3) $$\begin{array}{c} Z(\beta =1)=1. \end{array}$$

Of course, Equation (2) describes the size of the multifractal substrate or company market, while Equation (3) comes from the normalization condition of shares.

Moreover, using the share limitation from below and above, we get

(4) $$\begin{array}{c} Z\left(\beta \to \mp \infty \right)\approx \left\{\begin{array}{c} {\left(\omega^{\min}\right)}^{\beta },\;\beta <0\\ {\left(\omega^{\max}\right)}^{\beta },\;\beta >0 \end{array}\right. \end{array}$$

where $\omega^{\min}$ and $\omega^{\max}$ determine the marginal values of the companies’ market shares.

### 2.2. Scaling Relations

We continue to show that the partition function Z(β) takes the form of a power law,

(5) $$\begin{array}{c} Z\left(\beta \right)=\Lambda^{-\tau (\beta )}\Leftrightarrow \tau \left(\beta \right)=-\frac{\ln Z(\beta )}{\ln\Lambda }, \end{array}$$

where τ(β) is the scaling exponent, while the base/scale Λ we define below. Having the partition function at our disposal, we can build a thermodynamic formalism on this basis. We talk more about it in Section 2.5, where we calculate a specific heat.

To prove the correctness of the first equality Equation (5), we use two crucial scaling exponent properties,

(6) $$\begin{array}{c} \tau (\beta )=(\beta -1)D(\beta ) \end{array}$$

where D(β)≥0 is the Rényi dimensions and

(7) $$\begin{array}{c} \tau (\beta )=\beta h(\beta )-D(\beta =0), \end{array}$$

here h(β) is a generalized Hurst exponent and D(β=0) is the Hausdorff dimension of the substrate/market, which for our case we can put to 1.

For β→1 the Rényi information approaches the Shannon information that is, it becomes the information dimension,

(8) $$\begin{array}{c} D\left(\beta =1\right)=-\frac{1}{\ln N}\sum_{n=1}^{N}\omega_{n}\ln\omega_{n}. \end{array}$$

For β→2 the partition function (1) reduces to the well-known correlation integral C(N) of Grassbereger and Procaccia [16], i.e.,

(9) $$\begin{array}{c} D\left(\beta =2\right)=-\frac{\ln C(N)}{\ln N}. \end{array}$$

Furthermore, let us also note that always $D\left(\beta^{\prime }\right)\leq D\left(\beta \right)$ for $\beta <\beta^{\prime }$.

Now, we can define basis Λ. We use Equation (5) for this purpose, in which we put β=0 followed by Equations (2) and (7). Therefore, we get Λ=N.

The above result, in combination with the scaling Equation (5), allow us to present the scaling exponent in an explicit asymptotic form,

(10) $$\begin{array}{c} \tau \left(\beta \to \mp \infty \right)\approx \left\{\begin{array}{c} -\beta \frac{\ln\omega^{\min}}{\ln N},\;\beta <0\\ -\beta \frac{\ln\omega^{\max}}{\ln N},\;\beta >0. \end{array}\right. \end{array}$$

**Figure 3:**
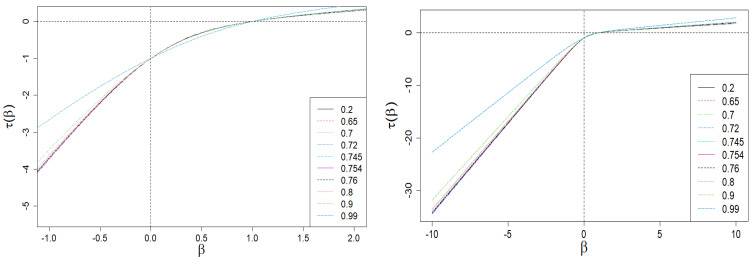
Scaling exponent τ(β) vs. exponent β (the order of scale). Its nonlinear/multifractal behavior in the range of β∈[−1.0,2.0] for interventionism level 0<q<1 is clearly seen (especially on the zoomed plot). On the other hand, the plot on the right shows the existence of oblique asymptotes. Multifractality is present if and only if they are different from each other. For example, we have selected ten characteristic levels of interventionism here (see the legend). The sharp decrease in the slope difference of the asymptotes for q≈1 (blue dashed curves) is visible. We use the same set of *q* values in all plots in Section 2.

With the above results, we can now present a plot of τ(β) vs. β—this plot and its enlarged version limited to the central values of β (from the range of [−1.5,1.5]), are presented in Figure 3. As one can see, τ(β) is bounded by two diagonal asymptotes defined by Equation (10).

We consider the next two extreme cases. The first, is when all but one of the company shares disappear (the case of a monopolized market). Then, with Equations (1), (3) and (5), we get immediately that τ(β) is undefined.

The second case is when all shares are equal (the case of the egalitarian market), i.e., $\omega_{n}=\frac{1}{N},\;n=1,2,\ldots ,N$. Then, with Equations (1), (3) and (5), we get

(11) $$\begin{array}{c} \tau (\beta )=\beta -1, \end{array}$$

i.e., the scaling exponent is a linear function of β. We continue to deal mainly with cases distant from both of the above extreme cases.

We assume that company shares, $\omega_{n}$, create the nonuniform/multiscale function $\omega_{n}$ vs. *n*, a multifractal structure. In other words, we are dealing here with multifractality, the source of which is the heterogeneous distribution of company shares.

### 2.3. Rényi Dimensions and Generalized Hurst Exponent

**Figure 4:**
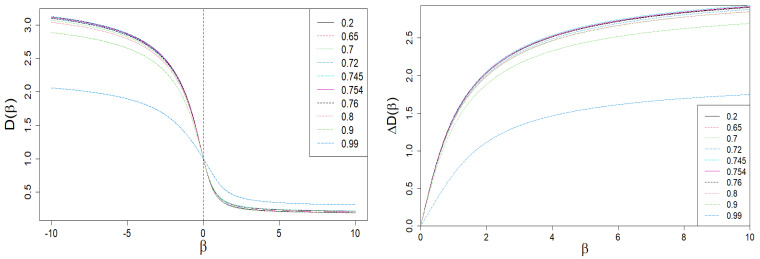
Dependence of Rényi dimensions *D* on β. A sharp drop in the ΔD(β) span is clearly visible on the right plot for large values of |β| and q≈1 (blue dashed curve). This is the result of the behavior of the τ(β) vs. β curve shown in Figure 3.

**Figure 5:**
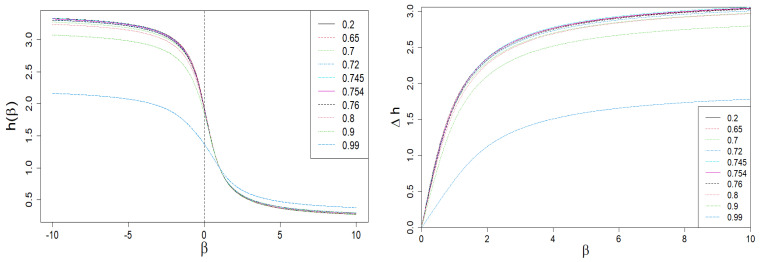
The dependence of the generalized Hurst exponent *h* and its span Δh on β. A sharp drop in the Δh(β) span is clearly visible for large values of |β| and q≈1 (blue dashed curve). It is the result of the behavior of the τ(β) vs. β curve shown in Figure 3.

In Figure 4 and Figure 5, we present the Rényi dimensions, D(β), generalized Hurst exponent, h(β), and their spans ΔD(β)=D(−β)−D(β) and Δh(β)=h(−β)−h(β), respectively. The former two quantities are limited by identical horizontal asymptotes:

(12) $$\begin{array}{ccc} & & D(\beta \to \mp \infty )=h(\beta \to \mp \infty )\\ & = & \left\{\begin{array}{c} D^{\max}=h^{\max}=-\frac{\ln\omega^{\min}}{\ln N},\beta <0,\\ D^{\min}=h^{\min}=-\frac{\ln\omega^{\max}}{\ln N},\beta >0, \end{array}\right. \end{array}$$

while

(13) $$\begin{array}{ccc} & & \Delta D\left(\beta \to \infty \right)=D^{\max}-D^{\min}\\ & = & \Delta h\left(\beta \to \infty \right)=h^{\max}-h^{\min}\\ & = & \ln\left(\frac{\omega^{\max}}{\omega^{\min}}\right). \end{array}$$

Equations (12) and (13) are a direct result of the asymptotic scaling exponent properties given by Equation (10) and by Equations (6) and (7), respectively.

### 2.4. Spectrum of Dimensions

We now designate the most crucial multifractality signature, i.e., the spectrum of dimensions (singularities), *f*, given by the Legendre transformation,

(14) $$\begin{array}{c} f(\alpha )=\beta (\alpha )\alpha -\tau (\beta (\alpha )), \end{array}$$

where the local dimension (singularity or Hölder exponent) is

(15) $$\begin{array}{c} \alpha \left(\beta \right)=\frac{d\tau (\beta )}{d\beta }=-\frac{1}{\ln N}\frac{\sum_{n}\omega_{n}^{\beta }\ln\omega_{n}}{\sum_{n}\omega_{n}^{\beta }}. \end{array}$$

Therefore, we obtain a helpful equality locating the maximum spectrum of dimensions f(α(β=0)),

(16) $$\begin{array}{c} \alpha \left(\beta =0\right)=-\frac{1}{N\ln N}\sum_{n}\ln\omega_{n}. \end{array}$$

and we get, analogously as in Equation (12),

(17) $$\begin{array}{c} \alpha \left(\beta \to \mp \infty \right)\approx \left\{\begin{array}{c} \alpha^{\max}=-\frac{\ln\omega^{\min}}{\ln N},\\ \alpha^{\min}=-\frac{\ln\omega^{\max}}{\ln N}. \end{array}\right. \end{array}$$

As one can see from Equation (12), the quantities D,h, and α have the same lower and upper bounds.

Furthermore, from Equations (14) and (15) we get

(18) $$\begin{array}{c} \beta =\frac{df(\alpha )}{d\alpha }. \end{array}$$

**Figure 6:**
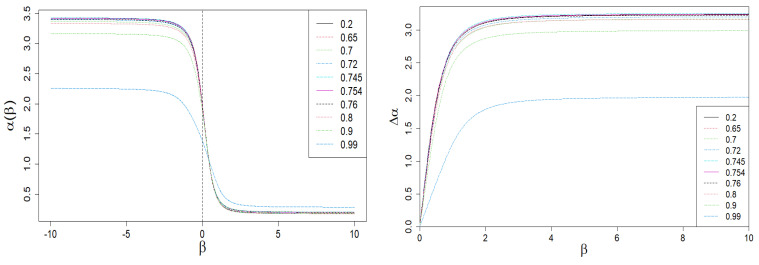
Dependence of the local singularity α on β. A sharp drop in the Δα(β) span is clearly visible on the right plot for large values of |β| and q≈1 (blue dashed curve). This is the result of the behavior of the τ(β) vs. β curve shown in Figure 3.

In Figure 6, we present the dependence of local exponent α and its span Δα on β.

**Figure 7:**
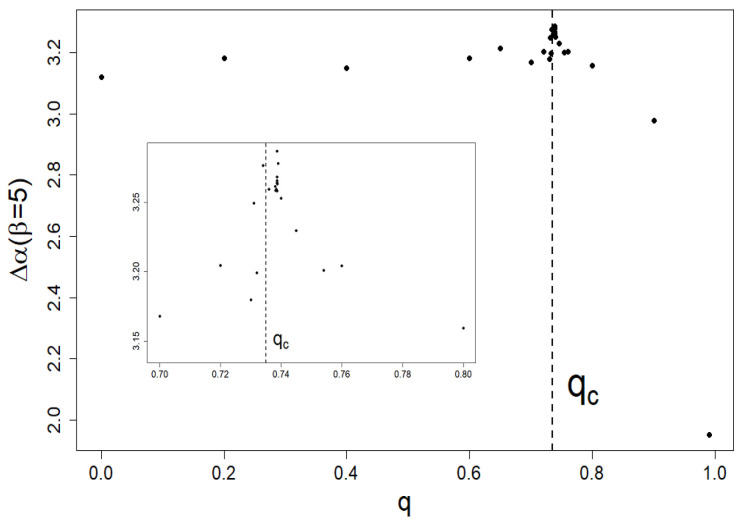
Dependence of the local singularity span Δα on *q* at fixed β=5. A slight but distinct peak locates near $q_{c}=0.735$, which defines the criticality threshold used by us at earlier work [15]. We also included a magnification of this peak.

In Figure 7, we present the dependence of the local singularity span Δα on *q* at fixed β=5.0.

**Figure 8:**
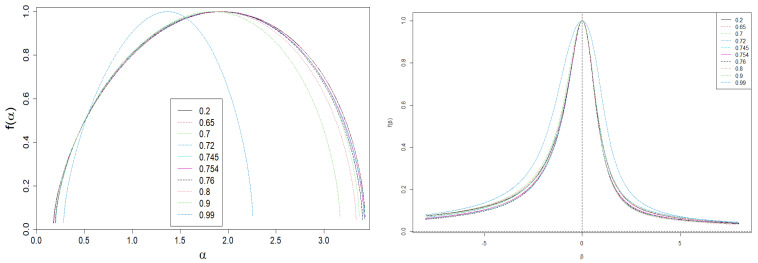
Dependence of spectrum of dimensions, *f*, from α (left plot) and β (right plot). There is a visible nonlinear dependence of the shape *f* on the level of interventionism *q*. Moreover, there is a wide spread in the spectrum of singularities Δα. As expected, the same applies to the dependence of *f* on β. In addition, there is a slight asymmetry of *f*, i.e., γ>0, herein.

Figure 8 shows he dependencies of *f* on α and on β. The α(β) vs. β plot (like D(β) and h(β) vs. β ones) is limited by two horizontal asymptotes given by Equation (17). This is a direct result of the asymptotic properties of Equation (10).

We present below useful quantities, which characterize the spectrum of singularities:

(i) $f^{0}=f\left(\alpha \left(\beta =0\right)=\alpha^{0}\right)=D^{0}=D\left(\beta =0\right)$, which results from Equations (7), (14) and (18), and moreover we get $\frac{df(\alpha )}{d\alpha }{|}_{\beta =0}=0$;
(ii) for β=1 we immediately get from Equation (18) $\frac{df(\alpha )}{d\alpha }{|}_{\beta =1}=1$, therefore $f^{1}=f\left(\alpha \left(\beta =1\right)\right)=\alpha \left(\beta =1\right)=\alpha^{1}$;
(iii) for β→−∞ we get from Equations (14) and (15), that $f^{\max}=f\left(\alpha =\alpha^{\max}=-\frac{\ln\omega^{\min}}{\ln N}\right)$ = 0 and $\frac{df(\alpha )}{d\alpha }{|}_{\beta \to -\infty }=-\infty$; similarly for β→∞ we get $f^{\min}=f\left(\alpha =\alpha^{\min}=-\frac{\ln\omega^{\max}}{\ln N}\right)$ = 0 and $\frac{df(\alpha )}{d\alpha }{|}_{\beta \to \infty }=\infty$;
(iv) the maximum span of *f* we determine as follows, ${\Delta \alpha |}_{|\beta |\to \infty }=\alpha^{\max}-\alpha^{\min}=\frac{1}{\ln N}$$\ln\left(\frac{\omega^{\max}}{\omega^{\min}}\right)$. We continue to use the simplified designation ${\Delta \alpha =\Delta \alpha |}_{|\beta |\to \infty }$;
(v) the following asymmetry factor can be used to determine the degree of asymmetry *f*, ${\gamma |}_{|\beta |\to \infty }=\frac{\alpha \left(\beta =0\right)-\alpha^{\min}}{\alpha^{\max}-\alpha \left(\beta =0\right)}$, where α(β=0) is given by Equation (16). We continue to use the simplified designation ${\gamma =\gamma |}_{|\beta |\to \infty }$.

It should be emphasized that in general $f(\alpha =\alpha^{\min},\alpha^{\max})\neq 0$. This happens when at least one of the boundary values $\omega^{\min},\omega^{\max}$ is degenerated. This is discussed in Section 3.2 for a four-group company market model.

The large span Δα visible in Figure 8 indicates a great volatility of competing firms on the market. At the same time, we deal with a wide variety of companies only when it also occurs that N≫1. However, the shift of the spectrum of dimensions to higher values of α signals the dominance of smaller companies on the market. Let us note that we would deal with a weak multifractality if and only if the span Δα≪1.

One can also analyze asymmetry of *f* using the coefficient γ. If γ>1, then we are talking about the advantage on the market of large companies, as opposed to the situation of γ<1. The marginal case γ=1 corresponds to the balanced situation.

### 2.5. Specific Heat

We can now define the specific heat *c* of the system/market on the reciprocal of the temperature β, as follows [4,14,17]:

(19) $$\begin{array}{ccc} c(\beta ) & = & -\beta^{2}{\left(\frac{\partial^{2}\left(\beta F/V\right)}{\partial \beta^{2}}\right)}_{V}\\ & = & \frac{1}{\ln N}\beta^{2}{\left(\frac{\partial^{2}\ln Z}{\partial \beta^{2}}\right)}_{N}, \end{array}$$

where $\frac{1}{V}\beta F=-\frac{1}{\ln N}\ln Z$, while *F* is the free energy of a company market, and V=lnN here.

**Figure 9:**
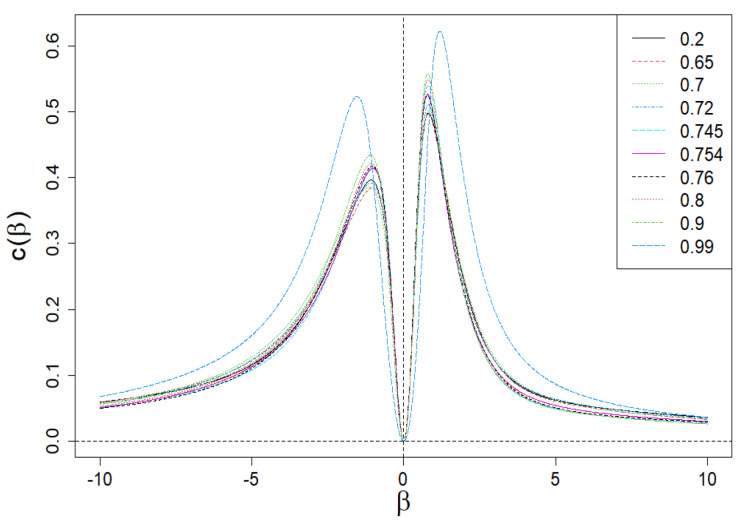
Dependence of specific heat, *c*, for a constant volume ($V=\ln N_{st}$) on β. The anomalous behavior of *c* is apparent due to the presence of Schottky peaks for both the positive and negative values of β.

The dependence of c(β) on β is presented in Figure 9. Apparently, this dependence is anomalous (both for positive and negative values of β) because it has a local peak, analogous to the Schottky peak for the specific heat of the solid [18,19] related to its internal degrees of freedom. Let us add that the disappearance of c(β=0) in β=0 results directly from the second formula (19). Such clear peaks are the result of highly differentiated values of the shares, $\omega_{i}$, that define partition function Z. They play the role of internal degrees of freedom here. We proof that Z composed of only two different shares $\omega^{\max}$ and $\omega^{\min}$ already leads to the anomalous peaks of specific heat.

## 3. Discussion and Concluding Remarks

### 3.1. Multifractality of Real Company Market

As an example of the method’s application, we exploit the ‘S&P500 Companies by Weight’ page (from the day 12 November 2021). (The data was taken from the page https://www.slickcharts.com/sp500. Accessing to this page is common and unlimited all the time). The available empirical data covers approximately 70–80% of the total US stock market capitalization. These empirical data directly provide the market daily share values of individual companies, i.e., the data we need.

**Figure 10:**
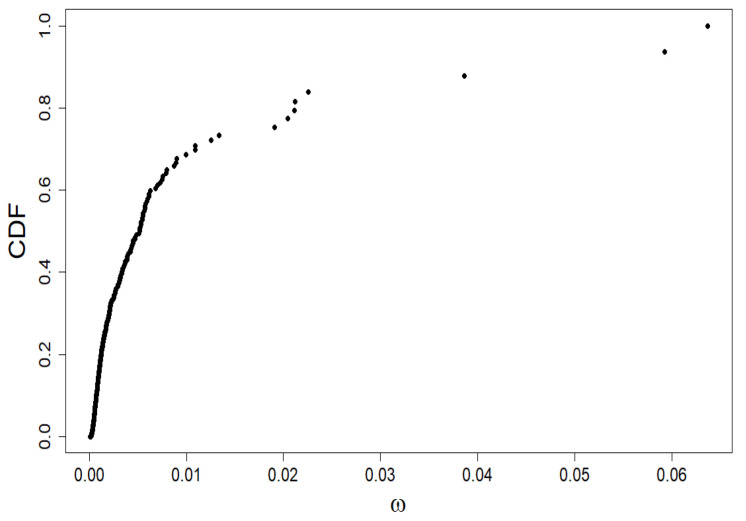
Quetelet curve: the empirical dependence of the standardized rank of companies, belonging to the S&P500 index, i.e., CDF, on their shares ω. It is precisely to analyze this data that we use multifractal formalism.

Let us characterize the market shares of companies using the Quetelet ranking (see Figure 10), i.e., we build a cumulative distribution function (CDF) versus company share value plot. The market structure is visible:

- the market segmentation into the overwhelming majority of companies with a small market share (around 0.01 or less)
- five companies with a market share between 0.02 and 0.03
- three companies with the highest market share between 0.04 and 0.065.

In such a situation, the question of the actual dominance of companies on the market is justified: will small companies dominate large ones, or is the opposite case. For this purpose, we use the multifractal analysis described in Section 2.

It is worth realizing that if the CDF was built on a power, exponential, or Gaussian distribution, we would not be dealing with multifractality. In the first case, the scaling exponent τ(β) would be a linear function of β, in the second case it would be logarithmic, and in the third case, it would be a linear combination of logarithmic and linear functions.

We continue to investigate the empirical relationship shown in Figure 10 with the multifractality approach shown in Section 2. When using Equations (1) and (5), we find the relationship τ(β) vs. β, but we do not go into whether the market is in a steady-state or not, i.e., the number of firms in the index $N=N\left(t\right)\neq N_{st}$ may fluctuate around 500 and shares may depend on time. We can use it here because the above considered method applies to both stationary and non-stationary states.

**Figure 11:**
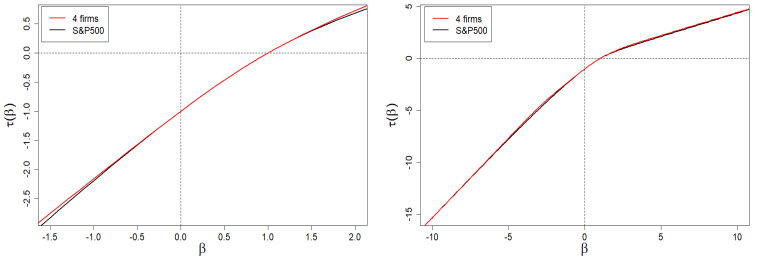
Dependence of τ(β) vs. β for the company market from the S&P 500 index. The left plot is a magnification of the β range belonging to the [−1.5,2.0] interval. The right plot shows the one in the full β range, i.e., belonging to the [−10,10] interval. In the assumed plot’s resolution of the whole (right) graph, it is impossible to distinguish the results of the four-group company market model (red curve) from the empirical (black) curve.

The above-mentioned relationship, τ(β) vs. β, is shown in Figure 11. The presented dependence is a nonlinear function of β, which allows us to carry out the next steps of the method.

**Figure 12:**
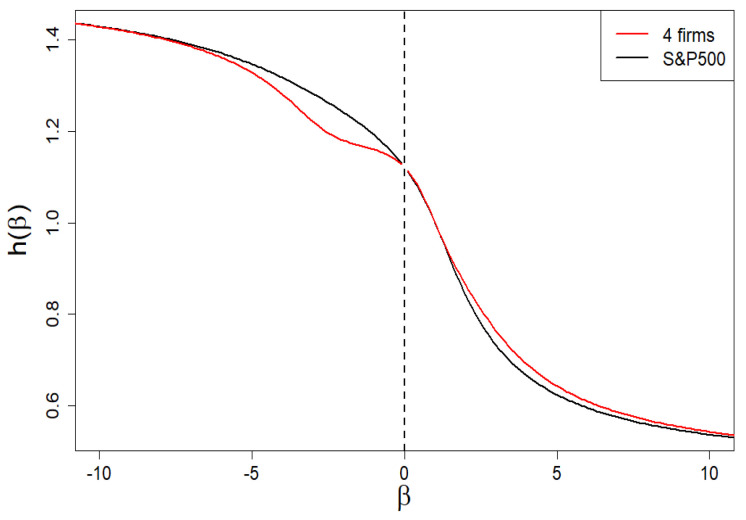
Dependence of the generalized Hurst exponent h(β) on the β exponent. Its span is sufficient for one of the spectra of dimensions presented in Figure 13 (both curves have there a common span) to define a solid multifractality. There are slight/subtle local differences between the two curves in both figures (black: the empirical one; red: the four-group company market).

**Figure 13:**
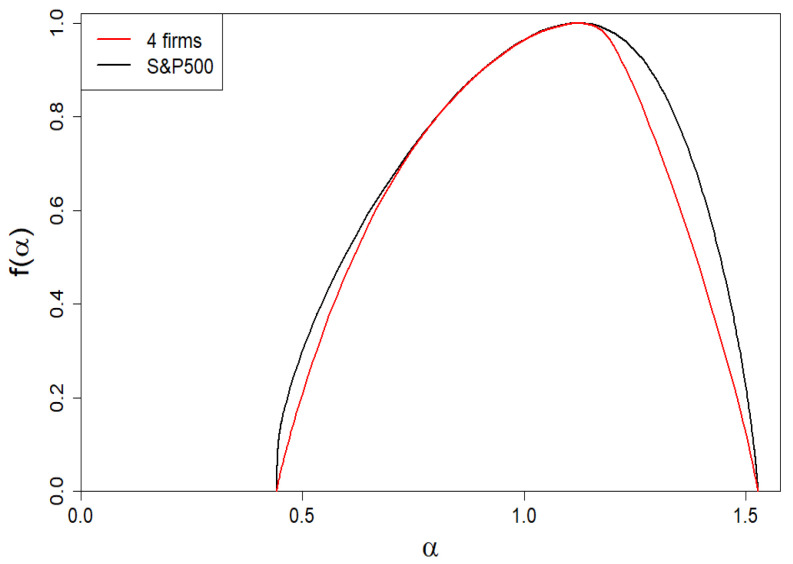
Dependence of the spectrum of dimensions f(α) vs. α for the company market from the S&P500 index (black curve). The *f* asymmetry favoring large firms is visible. For comparison, we have included the spectra of dimensions for the four-group company market represented by the red curve.

In Figure 12, we presented the dependence of the generalized Hurst exponent on the β exponent. Its span is sufficient for the one of the spectra of singularities presented in Figure 13 (cf. the black curve) to define a solid multifractality.

**Figure 14:**
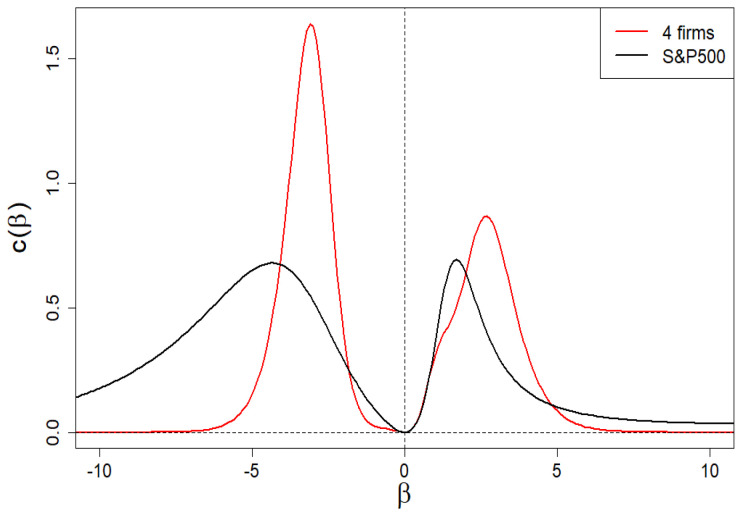
Anomalous dependence of specific heat c(β) vs. β for the company market, for example, from S&P500 index. As can be seen, the model of four-group company market shows apparent differences from the empirical data only at the level of the second τ derivative, i.e., at the level of hyper-fine effects.

In Figure 14, we show the specific heat c(β) vs. β. As in Section 2.5, we see peaks analogous to the Schottky peak—for both positive and negative values of β. There are differences in the predictions of the approach described below in Section 3.2 (in red) from the empirical curve (in black). These are hyper-fine deviations, as they appear at the level of the second order derivative of the scaling exponent τ.

We remind that subtle deviations (of the first order, i.e., at the level of the first derivative) are observed for the Hurst exponent as well as spectral dimension *f* (Figure 12 and Figure 13, respectively). Deviations regarding the τ curve itself are imperceptible (on the scale of the right plot in Figure 11).

### 3.2. Real Market vs. Four-Group Company Market

**Figure 15:**
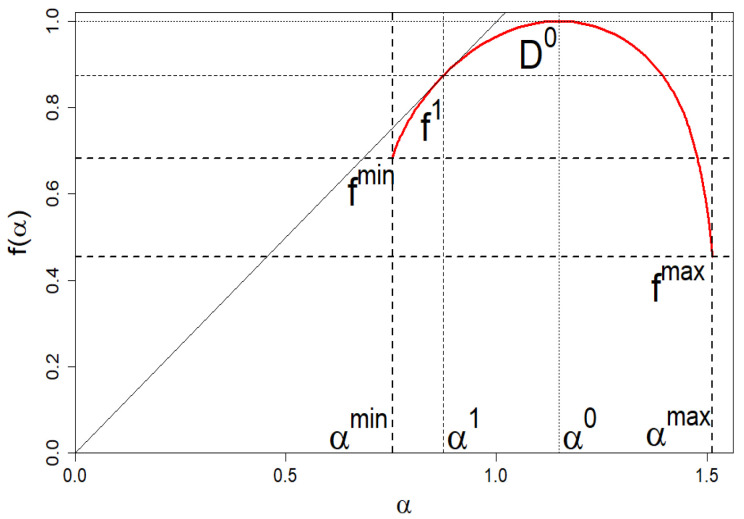
An example plot of the spectrum of dimensions *f* vs. α for the company market consisting of the four groups. Characteristic coordinates that we read from the graph, define the conditions (considered in the main text), which help us to determine the unknowns $M,K_{1},K_{2},L$ and $\omega^{\min},\omega_{1},\omega_{2},\omega^{\max}$.

Now, we answer the question: how should the market of companies be grouped/organized in order not to violate its diversity, i.e., to recreate its empirical spectrum of dimensions presented in Figure 13 (black curve). It is about its location and the basic shape defined by $\left(\alpha^{\min},f^{\min}\right),\left(\alpha^{1},f^{1}\right),\left(\alpha^{0},D^{0}\right)$, and $\left(\alpha^{\max},f^{\max}\right)$ (see Figure 15 for details).

We use for this purpose the following expression for the scaling exponent (based on the multifractal formalism presented in Section 2),

(20) $$\begin{array}{ccc} & & \tau \left(\beta \right)=-\frac{\ln Z_{4}\left(\beta \right)}{\ln N}=-\frac{1}{\ln N}\\ & \times & \ln\left(M{\left(\omega^{\min}\right)}^{\beta }+K_{1}\omega_{1}^{\beta }+K_{2}\omega_{2}^{\beta }+L{\left(\omega^{\max}\right)}^{\beta }\right), \end{array}$$

where $Z_{4}\left(\beta \right)$ means the partition function obtained from Equation (1) for the four-group company market. This section shows that such a division is enough to recreate the localization and shape of the spectrum of dimensions and other multifractality characteristics such as the scaling exponent, Hurst exponent, local exponent, and specific heat. We can show that two- and three-group company markets are not suitable for describing the multifractality of real company markets. For example, they cannot reproduce a location or a span of the spectrum of dimensions correctly.

Our specific goal is to clearly determine eight unknowns: the size of each of the four groups of companies $M,K_{1},K_{2},L$ and their shares $\omega^{\min},\omega_{1},\omega_{2},\omega^{\max}$. At least for the four-group company market, we can unambiguously determine the eight wanted unknowns.

Figure 15 shows an example schematic image of spectrum of dimensions—reading the coordinates of some of these points from this spectrum of dimensions allows us to determine the variables we are looking for. We show how to practically do this below.

The normalization condition takes the form

(21) $$\begin{array}{c} Z_{4}\left(\beta =1\right)=M\omega^{\min}+K_{1}\omega_{1}+K_{2}\omega_{2}+L\omega^{\max}=1, \end{array}$$

while the size of the market is fixed,

(22) $$\begin{array}{c} Z_{4}\left(\beta =0\right)=M+K_{1}+K_{2}+L=N. \end{array}$$

The point is that *N* is fixed either as a stationary value or an instantaneous value of the number of firms in the market. Therefore, we take it from empirical data.

We emphasize that Equations (21) and (22) are the first two equations from the system of equations that allow us to find the above-mentioned unknowns we are looking for. Because the shares of $\omega^{\min}$ and $\omega^{\max}$ are read directly from the empirical data, in order to find the remaining unknowns, we need four more equations, which we consider below.

From Equation (20), and Definitions (5) and (15), we get

(23) $$\begin{array}{ccc} \alpha (\beta ) & = & \frac{d\tau (\beta )}{d\beta }=-\frac{1}{\ln N}\frac{1}{Z_{4}\left(\beta \right)}\\ & \times & [M{\left(\omega^{\min}\right)}^{\beta }\ln\omega^{\min}+K_{1}\omega_{1}^{\beta }\ln\omega_{1}\\ & + & K_{2}\omega_{2}^{\beta }\ln\omega_{2}+L{\left(\omega^{\max}\right)}^{\beta }\ln\omega^{\max}] \end{array}$$

From the definition of the spectrum of dimensions (14), we obtain its boundary values for our case,

(24) $$\begin{array}{c} f^{\min}=f\left(\alpha^{\min}=\alpha \left(\beta \to \infty \right)\right)=\frac{\ln L}{\ln N},\\ f^{\max}=f\left(\alpha^{\max}=\alpha \left(\beta \to -\infty \right)\right)=\frac{\ln M}{\ln N}, \end{array}$$

which can also be read (to good approximation) from the empirical *f* shown in Figure 13 (black curve). Thus, the number of unknowns is reduced to two, namely, to $\omega_{1}$ and $\omega_{2}$. It should be emphasized that only in the special case, when *M* or *L* are equal to 1, i.e., when the marginal values of companies’ market shares are non-degenerate, do the boundary values of the spectrum of dimensions (24) disappear. It happens precisely in the case of the empirical data we use here.

Another needed quantity, which we read from the empirical *f* shown in Figure 13 (black curve), is the location of the center of the peak *f* given by the formula,

(25) $$\begin{array}{ccc} & & \alpha \left(\beta =0\right)=-\frac{1}{N\ln N}\\ & \times & \left(M\ln\omega^{\min}+K_{1}\ln\omega_{1}+K_{2}\ln\omega_{2}+L\ln\omega^{\max}\right). \end{array}$$

The same applies to the point of contact f(α(β=1))=α(β=1). Therefore,

(26) $$\begin{array}{ccc} & & \alpha \left(\beta =1\right)=-\frac{1}{\ln N}\\ & \times & [M\omega^{\min}\ln\omega^{\min}+K_{1}\omega_{1}\ln\omega_{1}\\ & + & K_{2}\omega_{2}\ln\omega_{2}+L\omega^{\max}\ln\omega^{\max}]. \end{array}$$

Both of the above equations have been obtained from Equation (20) and definition (15).

Now we calculate unknowns $K_{1}$ and $K_{2}$ from Equations (21) and (22) as the function of $\omega_{1}$ and $\omega_{2}$. We substitute the obtained quantities into Equations (25) and (26). Thus, we reduce our problem to two transcendental equations. For our case, M=L=1, these equations can be converted to the form

(27) $$\begin{array}{ccc} & & \alpha \left(\beta =0\right)N\ln N+\ln\left(\omega^{\min}\omega^{\max}\right)\\ & = & \left(N-2\right)\frac{\omega_{1}\ln\omega_{2}-\omega_{2}\ln\omega_{1}}{\omega_{2}-\omega_{1}}+\Omega \frac{\ln\left(\frac{\omega_{1}}{\omega_{2}}\right)}{\omega_{2}-\omega_{1}}, \end{array}$$

and

(28) $$\begin{array}{ccc} & & \alpha \left(\beta =1\right)\ln N+\omega^{\min}\ln\omega^{\min}+\omega^{\max}\ln\omega^{\max}\\ & = & \left(N-2\right)\frac{\omega_{1}\omega_{2}}{\omega_{2}-\omega_{1}}\ln\left(\frac{\omega_{2}}{\omega_{1}}\right)+\Omega \frac{\omega_{1}\ln\omega_{1}-\omega_{2}\ln\omega_{2}}{\omega_{2}-\omega_{1}}, \end{array}$$

(where $\Omega =1-\omega^{\min}-\omega^{\max}$), which are more convenient for a numerical solution. Thus we have reduced our problem to the above two transcendental equations.

Table 1 presents the empirical data needed here regarding the first and last components of the S&P500 index of 12 November 2021, consisting (on this day) of N=505 companies.

Based on these empirical data, we solve numerically Equations (27) and (28) and obtain $\omega_{1}=0.00065$ and $\omega_{2}=0.0101$. Therefore, we have $K_{1}=439$ and $K_{2}=64$. Thus, in our case, we obtain non-degenerate share margins and strongly degenerate (though very different) intrinsic share values. The resulting spectrum of dimensions we presented in Figure 13 by means of a red curve. Likewise, we have presented the remaining results in Figure 11, Figure 12 and Figure 14 by means of red curves.

We emphasize that the obtained result is universal in the sense that, starting from the four-group market of companies, we obtain enough equations to describe the location and shape of the multifractality characteristics.

### 3.3. Conclusions

It is worth realizing how distributions induce common multifractal structures. Therefore, it is not so much about searching for such structures, but about the possibility of comparing them with each other, i.e., answering the question of which structures are more multifractal and which are less. For this, they must first be classified according to their symmetry and degeneration. The larger the logarithm of these steps, the higher these elevations are.

The degree of asymmetry in the multifractal structure is determined by the γ asymmetry coefficient. If γ=1, we have a symmetric multifractal structure. If γ>1, we have left asymmetry, while for γ<1, we have right asymmetry.

The degree of degeneration of the marginal shares determines the elevation of the edges of the spectral dimensions: the left one depends on the degree of degeneration of the maximum share, and the right one depends on the degree of degeneration of the minimum share.

**Figure 16:**
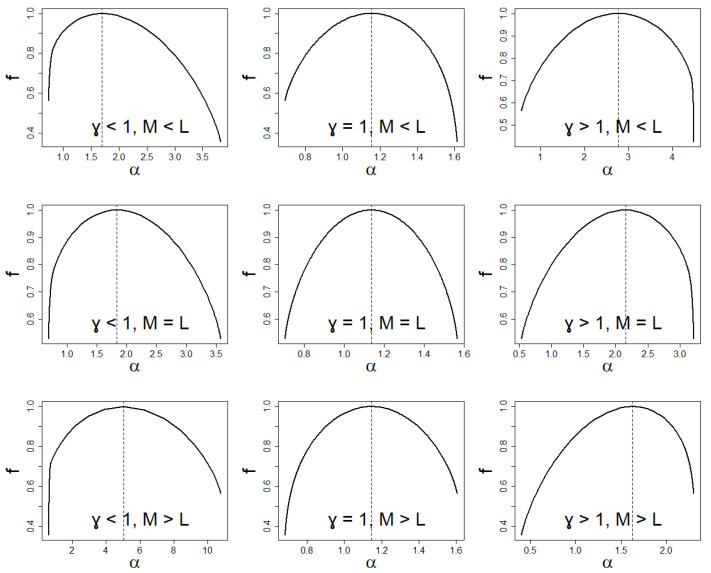
Schematic classification of spectrum of dimensions due to asymmetry γ and degeneration (M,L).

In this way, we have divided multifractal structures into nine groups, where both asymmetries and degenerations match themselves like the symmetry of the left and right hands (see Figure 16 for illustration, there, for example, the first plot in the first column and the last plot in the third column). Only within each group can we introduce a measure that allows us to organize the multifractal structure. The above classification is possible due to the fact that asymmetry and degeneration are independent of each other.

Suppose two multifractal structures have the same span of the spectrum of dimensions and location. One is more multifractal than the other if its degeneration levels are less than the corresponding other.

Another special case is when both multifractal structures’ degeneracy levels are equal, while the structures differ in span. Then the more multifractal structure is for, the larger span structure plus $f^{1}$.

We introduce a precise definition of the linear multifractal capacity, M, utilizing a definition based on Figure 15 and Equation (24),

(29) $$\begin{array}{c} M=\Delta \alpha +f^{1}+M^{-1}+L^{-1}. \end{array}$$

Notably, there is no differentiation of multifractality due to location $\alpha^{0}$. The proposed phenomenological measure of multifractal capacity, M, is a partial in the sense that it does not take into account the entire fine structure of the spectrum of dimension *f*.

In conclusion, in this paper, we examine the multifractality/multiscaling coming from shares and not from correlations. In this sense, this work is complementary to our previous one [15]. As a reference case, we have discussed the instructive example of the four-group company market. We have shown that (within the zero-order approximation) each market can be reduced to a four-group company market, which should facilitate market analysis.

Finally, we can say that this is the first time such a multifractal analysis of the market of competing companies has been performed.

Notably, we can apply the approach to any series of shares, e.g., shares of turnover volumes on the stock exchange and shares of companies’ quotations on the stock exchange. In short, the approach can be applied to any normalized series of positively defined elements. Moreover, our approach makes it possible to examine the evolution of multifractality of company market especially in the vicinity of crash regions. That is why it is so important to study in the near future the relationship between multifractality and criticality suggested by Figure 7.

## Figures and Tables

**Table 1:** Empirical data on the first and last components of the S&P500 index as of 12 November 2021.

No.	Company	$\omega^{\min}$	$\omega^{\max}$	*M*	*L*
1	AAPL (Apple Inc., Cupertino, CA, USA)	−	0.06866056	−	1
505	NWS (New Corporation Class B, New York, NY, USA)	0.00006948	−	1	−

## Data Availability

Data available in a publicly accessible repository. The data presented in this study are openly available in “*S*&*P* 500 Companies by Weight” at https://www.slickcharts.com/sp500.
